# Long non-coding RNA AC012668 suppresses non-alcoholic fatty liver disease by competing for microRNA miR-380-5p with lipoprotein-related protein LRP2

**DOI:** 10.1080/21655979.2021.1960463

**Published:** 2021-09-13

**Authors:** Xiaomeng Chen, Hong Ma, Yan Gao, Ye Jin, Wei Ning, Yue Hou, Jianrong Su

**Affiliations:** a Clinical Laboratory Center, Beijing Friendship Hospital, Capital Medical University, China; b Beijing Friendship Hospital, Capital Medical University, Beijing, China

**Keywords:** Nonalcoholic fatty liver disease, long non-coding RNA, miR-380-5p, LRP2, hepatic steatosis

## Abstract

Nonalcoholic fatty liver disease (NAFLD) is characterized by high morbidity. Although long noncoding RNAs (lncRNAs) are known to have a role in NAFLD pathogenesis, the identified lncRNA types are limited. In this study, NAFLD models were established in vitro and in vivo using free fatty acid-treated LO2 cells and high-fat diet-fed mice, respectively. Microarray data were downloaded from the Gene Expression Omnibus database, and *AC012668* was selected for further analysis. Cell viability and apoptosis were measured using Cell Counting Kit 8 and flow cytometry assays. RNA expression was detected using reverse transcription-quantitative polymerase chain reaction. Triglyceride (TG) content and lipid deposition were detected using enzyme-linked immunosorbent assay and Oil-Red O staining. Western blotting was used to visualize protein expression. Starbase and TargetScan were used to predict the target miRNA and gene, and the predictions were verified through RNA pull-down and luciferase reporter assays. *AC012668* expression levels were significantly suppressed in NAFLD models, whereas *AC012668* overexpression inhibited lipogenesis-related gene (*SCD1, SREBP1, FAS*) expression and TG/lipid accumulation in vitro. Subsequently, *miR-380-5p* was predicted and verified to target *AC012668*, and its expression was notably increased in the NAFLD cell model. Moreover, transfection of *miR-380-5p* antagonized the effects of *AC012668* on lipid formation and accumulation. *LRP2* was confirmed to be the target gene of *miR-380-5p* and was downregulated in the NAFLD cell model. Silencing *LRP2* reversed the effects of the *miR-380-5p* inhibitor on lipid formation and accumulation. *AC012668* inhibited NAFLD progression via the *miR-380-5p*/*LRP2* axis. These findings may provide a novel strategy against NAFLD.

## Introduction

Fatty liver, one of the most common liver disorders worldwide, is frequently caused by excessive intake of carbohydrates and lipids [1]. According to its pathogenesis, fatty liver is classified into two categories, alcoholic fatty liver disease and nonalcoholic fatty liver disease (NAFLD), of which NAFLD is more common [2]. NAFLD is a disease spectrum that progresses from simple hepatic steatosis to inflammatory responses, developing into nonalcoholic steatohepatitis (NASH) and hepatic fibrosis and ultimately leading to loss of liver function [3]. Hepatic steatosis refers to a triglyceride (TG) level in the liver exceeding the 95% percentile in healthy individuals or TG lipid droplets filling > 5% of the liver cytoplasm [4]. Simple hepatic steatosis is a self-limiting disease. NASH is considered present once hepatocellular damage, inflammation, or fibrosis occurs [5]. Approximately 10% to 29% of patients with NASH will progress to cirrhosis within 10 years [6]. At present, about 1 billion people worldwide have NAFLD [7]. However, the treatment of NAFLD remains unsatisfactory and therapeutic drugs based on the pathogenesis of the disease are lacking [6]. Therefore, the development of a novel and effective treatment for NAFLD is an urgent requirement to improve the life quality of patients.

Noncoding RNAs (ncRNAs) are crucial regulators of molecules that modulate biological processes [8]. Long ncRNAs (lncRNAs) are a type of ncRNAs with a length of > 200 nucleotides [9]. lncRNAs are required for many epigenetic processes, such as dose compensation, genomic imprinting, DNA methylation, histone modification, and chromatin remodeling [10]. Dysregulated lncRNAs are closely involved in the development of NAFLD [11]. For instance, high expression of the lncRNA *GAS5* is closely associated with the evolution from NAFLD to advanced liver fibrosis [12]. *HULC* knockdown inhibits the development of NAFLD [13]. Moreover, lncRNAs act as competing endogenous RNAs (ceRNAs) to regulate biological processes by sponging microRNAs (miRNAs). *NEAT1* deteriorates NAFLD by regulating the *miR-140*/*AMPK* axis [14]. *Gm12664-001* sponges *miR-295-5p* to induce the activation of *CAV1*, which alleviates NAFLD [15]. The lcnRNA *AC012668* is located in chromosome 2. However, the role of *AC012668* has not been previously elucidated. Low-density lipoprotein-related protein 2 (*LRP2*) is primarily expressed in absorptive epithelial tissues [16]. *LRP2* has a pivotal function in metabolic diseases [17]. To date, many lncRNA-miRNA pathways have been demonstrated to modulate NAFLD development [18,19]. Our group identified a novel lncRNA, *AC012668*, that is downregulated in NAFLD and functions as a sponge of *miR-380-5p* to promote the expression of *LRP2*.

Thereby, this study aimed to explore the role of AC012668 in NAFLD via establishing the model in vivo and in vitro. We hypothesized that *AC012668* may suppress the progression of NAFLD via the *miR-380-5p*/*LRP2* axis.

## Materials and methods

### NAFLD mouse model

Twenty 8-week-old male specific pathogen-free grade C57BL/6 J mice were purchased from Shanghai Southern Model Center. The mice were housed in a dry and ventilated pathogen-free barrier facility with 60% relative humidity, 12 h/day lighting, and 25°C temperature. The animals were randomly divided into two groups, 10 mice fed a regular diet and 10 mice fed a high-fat diet (HFD) for 12 weeks, to establish NAFLD models. The HFD (D12492, Beijing, China) consisted of protein, fat, and carbohydrates at 20%, 60%, and 20% of the total calories, respectively. The control group received a regular diet consisting of protein, fat, and carbohydrates at 20%, 10%, and 70% of the total calories, respectively. After 12 weeks of feeding, the mice were sacrificed through intraperitoneal injection of sodium pentobarbital (150 mg/kg) and the livers were collected for the subsequent experiments.

This study supervised by the Ethics Committee of Beijing Friendship Hospital, Capital Medical University Experimental Animal Center.

### Cell culture and transfection

Normal human hepatocyte lines LO2 were purchased from Biowit Biotechnology Inc. (cat no. C0009). The cells were maintained in Dulbecco’s modified Eagle’s medium containing penicillin, streptomycin (Macklin), and 10% fetal bovine serum (Ausbian) at 37°C. After 24 h, the cells were cultured with 1 mM free fatty acid (FFA; containing oleic acid and palmitic acid at a 2:1 volume ratio).

The cells were transfected with *AC120668*/*AC012668* small interfering RNA (*si-AC012668), miR-380-5p* mimic/inhibitor, and *si-LRP2* (NovaBio Co. Inc.) in Lipofectamine® 2000 reagent (Invitrogen). The negative controls were transfected with Lipofectamine 2000 alone.

### Microarray analysis

The microarray dataset GSE107231 was analyzed using Agilent-067406 Human CBC lncRNA + mRNA microarray V4.0. |logFC| > 2 and corrected P < 0.05 were used as standards. A total of 39 differentially expressed genes were screened, of which 18 were upregulated genes and 21 were downregulated genes. *AC012668* was selected for further analysis.

### Cell Counting Kit 8 (CCK8) assay

Thr CCK8 assay was conducted according to a previous study [20]. Cells were collected and resuspended at a density of 1 × 10^5^ cells/mL. Thereafter, the cell suspension was inoculated into 96-well plates at 100 μL/well. The cells were treated by adding 10 μL CCK8 reagent to each well and cultured at 37°C for 4 h. The absorbance values (450 nm) were obtained using a microplate reader (51119000, Thermo Fisher Inc.).

### Flow cytometric apoptotic assay

Apoptotic cells were stained using an Annexin V-fluorescein isothiocyanate (FITC) apoptosis detection kit and detected using Attune NxT Flow Cytometer and its supporting software (Thermo Fisher Inc.). The Annexin V-FITC reagent (5 μL) was added to the cells, and the apoptosis rates were determined using flow cytometry as described by Zan et al. [21].

### Oil-Red O staining assay

Oil-Red O staining assay was performed according to a previous study [22]. The LO2 cells were rinsed twice with phosphate-buffered saline (Meilunbio Biotechnology Co., Ltd) and placed on a coverslip after discarding the medium. The cells were fixed in 10% formaldehyde for 30 min. Oil-Red O (Solarbio) was added dropwise until the coverslip was completely covered. After 5 min of staining, the cells were washed with 60% isopropanol (Solarbio) and distilled water. Thereafter, the cells were counterstained with hematoxylin (Solarbio) for 2 min and observed under an optical microscope (SteREO Discovery.V20, Carl Zeiss Inc.).

### Enzyme-linked immunosorbent assay (ELISA)

The mouse livers were homogenized and centrifuged at 5000 rpm for 5 min, and the supernatant was collected for later use. The TG levels in the mouse livers were measured using an ELISA kit following the manufacturer’s protocol (SEKH-0380, Solarbio). The obtained values were normalized to the total protein levels. The hepatic TG level was expressed as μg/g protein. The total protein levels were measured using a bicinchoninic acid (BCA) protein assay kit (PC0020, Solarbio).

### Reverse transcription-quantitative polymerase chain reaction (RT-qPCR)

RNAs were collected from cells and tissues. RT-qPCR was performed using One Step SuperRT-PCR Mix Kit (T2240, Solarbio) on a Mastercycler® nexus (6330000072, Eppendorf Inc.). All primers used in the present study were designed and synthesized by Genewiz Inc. GAPDH and U6 served as loading controls. The thermocycling conditions were as follows: denaturation at 94°C for 60 s, annealing at 37°C for 60 s for 30 cycles, and extension at 72°C for 120 s. The results were analyzed using the 2^−ΔΔCt^ method [23]. The sequences of the primers were as follows: *AC012668* forward (F) 5'-ATCAGAATCACCTGGCGGTC-3', reverse (R) 5'-TGTACTAGCGGCATCAGCAG-3'; *SCD1* F 5''GCTGATCCTCATAATTCCCGA‐3', R 5';TTAAGCACCACAGCATATCGC‐3'; *SREBP1* F 5'-ACAGTGACTTCCCTGGCCTAT-3', R 5'-GCATGGACGGGTACATCTTCAA-3'; *FAS* F 5'-AAATGAAAGCCAACTGCATCGAC-3', R 5'-ATTGGACCCTCGCTGAGCAC-3'; *miR-380-5p* F 5'-CTCGCTTCGGCAGCACA-3', R 5'-CAGTGCGTGTCGTGGAGT-3'; *LRP2* F 5'-CCTTGCCAAACCCTCTGAAAAT-3'; R 5'-CACAAGGTTTGCGGTGTCTTTA-3'; and *GAPDH* F 5'-GGGAGCCAAAAGGGTCATCA-3', R 5'-TGATGGCATGGACTGTGGTC-3'.

### Western blotting assay

Western blotting assay was conducted according to previous study [24]. The total protein was collected and its level was determined using a BCA kit. The protein (40 μg) was separated on 10% sodium dodecyl sulfate-polyacrylamide gel electrophoresis gels for 1.5 h at 120 V, and the separated protein fractions were transferred onto polyvinylidine diflouride membranes (Millipore) for 2 h at 200 mA. The membranes were blocked with 5% fat-free milk for 1 h and incubated with primary antibodies, including anti-SCD1 (PAB15990, 1:1000; Abnova Biotech Inc.), anti-SREBP1 (3961–100, 1:1000; BioVision Inc.), anti-FAS (ANT-205, 1:500; Prospec Technologies Inc.), and anti-GAPDH (3777 R, 1:3000; BioVision), and with a secondary antibody (6916; 1:1000; BioVision). Finally, protein expression was analyzed using an electrochemiluminescence system.

### Luciferase reporter assay

Luciferase reporter assay was performed as described by Unal [25]. The wild and mutant type of *AC012668* and *LRP2* luciferase reporter vectors were designed and synthesized by Guangzhou RiboBio Co., Ltd. The wild-/mutant-type vectors and *miR-380-5p* mimic/control (RiboBio Co., Ltd.) were cotransfected into the LO2 cells, and the cells were incubated for 24 h. Thereafter, the cells were lysed to detect the luciferase activity using a TransDetect® Double-Luciferase Reporter Assay Kit (FR201-01, TransGen Biotech Co., Ltd.) 48 h after transfection.

### RNA pull-down assay

RNA pull-down assay was performed using an RNA pull-down kit (Bes5102, Bersin Biotechnology Co., Ltd.) according to a previous study [26]. Abiocenter (Beijing) Biotechnology Co., Ltd synthesized the biotinylated *miR-380-5p* probe and its control probe. Magnetic beads were resuspended in 50 μL RNA immunoprecipitation wash buffer and incubated with the biotin-labeled probes (50 pmol) at 4°C overnight. The cells were lysed with radioimmunoprecipitation assay buffer and RNase for 1 h to release the total RNA. Finally, the beads were washed six times with the lysis buffer. After the separation, the same RT-qPCR process as described above was used to quantify the relative expression of *LRP2* or *AC012668*.

### Statistical analysis

Data were analyzed using SPSS19.0 statistical software and expressed as mean ± standard deviation values. Student’s t test was used to assess the difference between two groups and analysis of variance (Duncan’s multiple range test) was applied for analyzing the data among multiple groups. P < 0.05 was deemed to indicate statistical significance.

## Results

Thereby, this study aimed to explore the effects of lncRNA *AC012668* on the TG accumulation and lipogenesis-related gene expression in NAFLD via establishing the model in vivo and in vitro. We demonstrated that lncRNA *AC012668* may suppress the progression of NAFLD via the miR-380-5p/LRP2 axis.

### lncRNA AC012668 expression level was decreased in HFD mice

**Figure 1.**
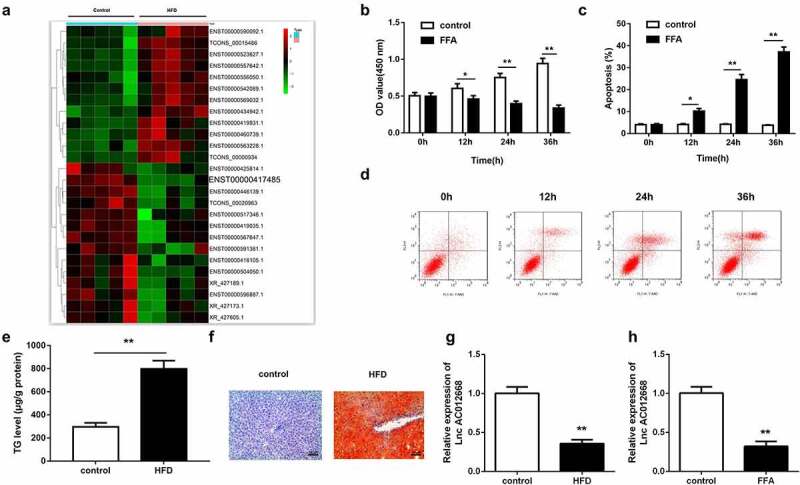
NAFLD models established to investigate changes in *AC012668* expression. (a) Heat map of differentially expressed lncRNAs in NAFLD. (b) Cell viability and (c and d) apoptosis of LO2 cells treated with FFA. (e and f) Oil-Red O staining image and TG level in the liver tissues of HFD mice. (g) *AC012668* expression levels in the liver tissues of HFD mice. (h) Expression of *AC012668* in FFA-treated cells. *P < 0.05 vs. control; **P < 0.01 vs. control

NAFLD, nonalcoholic fatty liver disease; TG, triglyceride; HFD, high-fat diet; FFA, free fatty acid.

Through microarray analysis, *AC012668* was predicted to be downregulated in NAFLD (Figure 1a). To further verify the role of *AC012668* in NAFLD, we generated FFA-treated LO2 cells and an HFD mouse model to simulate NAFLD in vitro and in vivo, respectively. FFA exposure significantly suppressed cell viability (Figure 1b) but significantly increased the apoptosis rate (Figure 1c and d), indicating the successful establishment of the NAFLD cell model. The results of both Oil-Red O staining and ELISA indicated that HFD significantly increased the TG content in the mouse livers (Figure 1e and f). RT-qPCR revealed that the *AC012668* expression level was downregulated in the in vivo and in vitro NAFLD models (Figure 1g and h).

### Overexpression of lncRNA AC012668 inhibited TG accumulation and lipogenesis-related gene expression in LO2 cells

**Figure 2.**
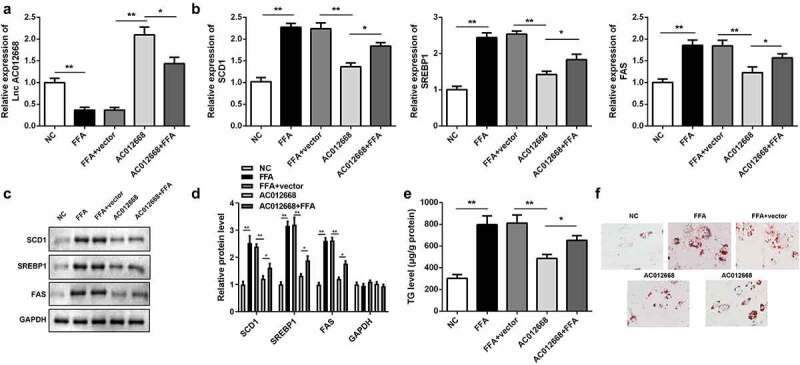
*AC012668* suppresses TG/lipid accumulation and lipogenesis in LO2 cells. (a) *AC012668* expression level in FFA-treated LO2 cells transfected with the *AC012668* overexpression plasmid. (b) mRNA and (c and d) protein expression levels of *SCD1, SREBP1*, and *FAS* in FFA-treated LO2 cells. (e) TG level and (f) Oil-Red O staining in FFA-treated LO2 cells. *P < 0.05 vs. *pcDNA3.1* or *AC012668.*

FFA, free fatty acid; TG, triglyceride.

Given the observed downregulation of *AC012668* in NAFLD, we further investigated the potential role of *AC012668* in vitro by transfecting cells with its overexpression plasmid. The *AC012668* overexpression plasmid significantly increased the expression of *AC012668* (Figure 2a). Additionally, *AC120668* suppressed the expression of lipogenesis-related genes, including *SCD1, SREBP1*, and *FAS*, in the FFA-treated cells in mRNA (Figure 2b) and protein levels (Figure 2c and d). Furthermore, the TG content was prominently reduced (Figure 2e) and lipid droplet deposition was ameliorated in the *AC012668* group (Figure 2f).

### lncRNA AC012668 targeted miR-380-5p

**Figure 3.**
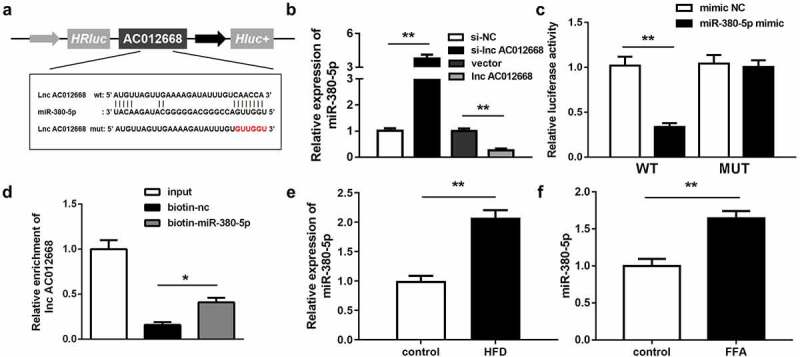
*miR-380-5p* directly targets *AC012668*. (a) Wild and mutant types of *AC012668* reporters labeled with luciferase. (b) Expression levels of *miR-380-5p* in *AC012668*-enhanced/-inhibited LO2 cells. (c) Relative luciferase activities in wild-type and mutant *AC012668* groups compared with the mimic negative control group. (d) Relative enrichment of *AC012668* in the biotinylated *miR-380-5p* group. (e) *AC012668* expression levels in the liver tissues of HFD mice. (f) Expression level of *miR-380-5p* in LO2 cells treated with 1 mM FFA. *P < 0.05 vs. biotin-NC; **P < 0.01 vs. si-NC, vector, mimic NC, or control

FFA, free fatty acid; NC, negative control; si-, small interfering RNA.

*AC012668* was predicted to target *miR-380-5p* in the analysis using Starbase (http://starbase.sysu.edu.cn/) (Figure 3a). The *miR-380-5p* expression level was significantly increased by *si-AC012668* and inversely inhibited by *AC012668* (Figure 3b). Cotransfection with the *miR-380-5p* mimic and *AC012668* (3′-untranslated region wild-type) markedly decreased the luciferase activity (Figure 3c). The RNA pull-down assay results further confirmed the interaction between *miR-380-5p* and *AC012668* (Figure 3d). In addition, FFA evidently promoted the expression of *miR-380-5p* in the in vivo and in vitro NAFLD models (Figure 3e and f).

### *miR-380-5p-facilitated lipogenesis antagonized lncRNA* AC012668

**Figure 4.**
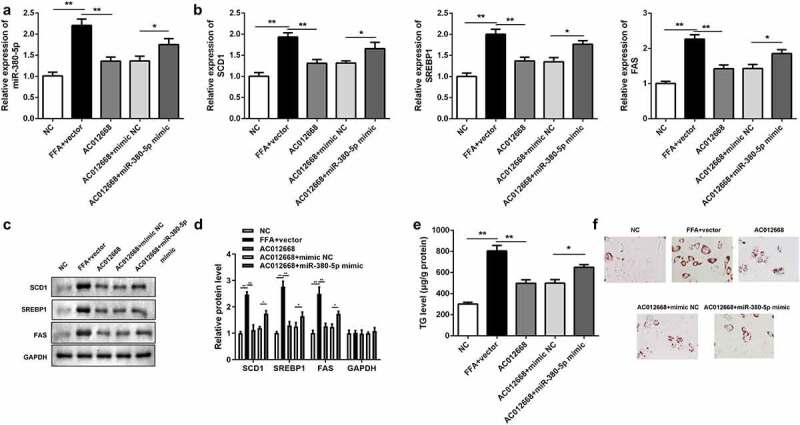
*miR-380-5p* promotes TG/lipid accumulation and lipogenesis in LO2 cells transfected with *AC012668*. (a) *AC012668* expression detected using reverse transcription-quantitative polymerase chain reaction. (b) mRNA and (c and d) protein levels of *SCD1, SREBP1*, and *FAS*. (e) TG level and (f) lipid deposition. *P < 0.05 vs. *AC012668* + mimic NC; **P < 0.01 vs. vector

TG, triglyceride; NC, negative control.

The effects of *miR-380-5p* on lipogenesis were also investigated. Cotransfection with the *miR-380-5p* mimic alleviated the effects of *AC012668* on the expression of *miR-380-5p* compared with the *AC012668* + negative control mimic group (Figure 4a). The inhibitory effects of *AC120668* on the expression of *SCD1, SREBP1*, and *FAS* were all partially reversed via the *miR-380-5p* mimic in the mRNA (Figure 4b) and protein levels (Figure 4c and d). Moreover, the TG content (Figure 4e) and deposition of lipid droplets (figure 4f) were increased by *miR-380-5p*.

### LRP2 was the downstream gene of miR-380-5p

**Figure 5.**
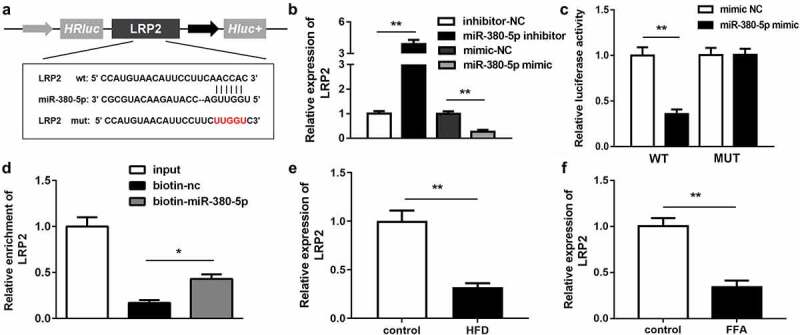
*LRP2* is a target of *miR-380-5p*. (a) Wild and mutant types of *LRP2* reporters labeled with luciferase. (b) mRNA expression of *LRP2* in FFA-treated LO2 cells transfected with the *miR-380-5p* mimic or inhibitor. (c) Relative luciferase activities in wild-type and mutant *LRP2* groups compared with the mimic negative control group. (d) Relative enrichment of *LRP2* in the biotinylated *miR-380-5p* group. (e) *AC012668* expression levels in the liver tissues of HFD mice. (f) Expression level of *LRP2* in FFA-treated LO2 cells. *P < 0.05 vs. biotin-NC; **P < 0.01 vs. inhibitor NC, mimic NC, or control

FFA, free fatty acid; NC, negative control.

*LRP2* was predicted to be the downstream target gene of *miR-380-5p* in the analysis using TargetScan (http://www.targetscan.org/mamm_31/) (Figure 5a). The *LRP2* expression level was significantly upregulated in the *miR-380-5p* inhibition group and downregulated in the *miR-380-5p* mimic group (Figure 5b). The RNA pull-down and luciferase reporter assay results further confirmed the interaction between *miR-380-5p* and *LRP2* (Figure 5c and d). In addition, the *LRP2* expression level was decreased in the in vivo and in vitro NAFLD models (Figure 5e and f).

### Silencing LRP2 neutralized the effects of miR-380-5p inhibitor on lipogenesis

**Figure 6.**
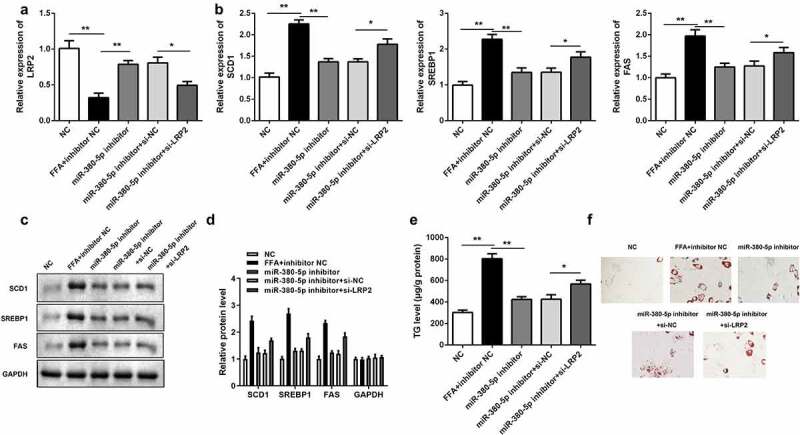
Effects of *LRP2* knockdown on TG/lipid accumulation and lipogenesis in *miR-380-5p*-inhibited LO2 cells. (a) *miR-380-5p* expression level in LO2 cells cotransfected with the *miR-380-5p* inhibitor and *si-LRP2*. (b) mRNA and (c and d) protein levels of *SCD1, SREBP1*, and *FAS* in the *miR-380-5p* inhibitor- and *si-LRP2*-cotransfected cells. (e) TG level and (f) lipid deposition in the *miR-380-5p* inhibitor- and *si-LRP2*-cotransfected cells. *P < 0.05 vs. control or *miR-380-5p* mimic + si-NC

TG, triglyceride; NC, negative control; si-, small interfering RNA.

*si-LRP2* reversed the upregulation of *LRP2* induced by the *miR-380-5p* inhibitor in LO2 cells (Figure 6a). Knockdown of *LRP2* antagonized the effects of *miR-380-5p* and significantly increased expression levels of lipogenesis-related genes (*SCD1, SREBP1, FAS*) in the mRNA (Figure 6b) and protein (Figure 6c and d) levels. Furthermore, *LRP2* knockdown increased the TG level (Figure 6e) and the amount of lipid deposition (Figure 6e and f).

## Discussion

NAFLD is the leading cause of chronic hepatopathy [27]. However, few targeted drugs have been clinically applied [28]. In the current study, we screened for downregulated lncRNAs in NAFLD. *AC012668* was first identified and selected for subsequent experiments. The expression of *AC012668* was inhibited in vitro and in vivo. In addition, FFA-induced lipogenesis and lipid deposition were alleviated by *AC012668. AC012668* upregulated *LRP2* by sponging *miR-380-5p*. Surprisingly, overexpression of *miR-380-5p* or silencing of *LRP2* reversed the effects of *AC012668* overexpression and promoted lipogenesis. These findings demonstrated the role of an *AC012668*/*miR-380-5p*/*LRP2* pathway in the pathogenesis of NAFLD.

Numerous studies have demonstrated that lncRNAs participate in the progression of NAFLD [29–31]. For instance, the lncRNA *NEAT1* was upregulated in NAFLD models and promoted lipid accumulation by targeting the *miR-146a-5p*/*ROCK1* axis [14]. Huang et al. [32] observed that overexpression of *MEG3* inhibited the process of lipogenesis via the *miR-21*/*LRP6* axis. Sponging of *miR-742-3p* by *Gm15622* enhanced the expression of the transcriptional regulator SREBP1c and promoted lipid accumulation in the NAFLD models [33]. Herein, we first documented that *AC012668* was downregulated in NAFLD in vivo and in vitro. Overexpression of *AC012668* reduced the expression of lipogenesis-related genes and the TG level. These inhibitory effects on lipogenesis suppressed the progression of NAFLD, consistent with the findings of previous studies [14,32,33].

lncRNAs function as ceRNAs to regulate gene expression through binding to miRNAs [11]. In this study, *miR-380-5p* was a target of *AC012668. miR-380-5p* was first reported to be highly expressed in the majority of primary neuroblastomas and to function as a proto-oncogene in a mouse mammary transplant model [34]. A microarray analysis study revealed that *miR-380-5p* was associated with metastasis and poor prognosis in breast cancer [35]. In this study, overexpression of *miR-380-5p* attenuated the effects of *AC012668* and promoted lipogenesis in LO2 cells, suggesting that *miR-380-5p* overexpression is closely related to the progression of NAFLD. Meanwhile, transfection of the *miR-380-5p* mimic exacerbated lipogenesis and lipid deposition, attenuating the effects of *AC012668*.

Previous studies mainly focused on the possible role of *miR-380-5p* in tumors [34,35]. However, its potential role in lipid metabolism has not been reported. In this study, *miR-380-5p* played a positive role in lipogenesis and enhanced the development of NAFLD from lipid metabolism disorders. These findings may add to the existing knowledge about *miR-380-5p*. Suppressing the expression of *miR-380-5p* may be an alternative method to suppress the progression of NAFLD.

LRP2, also known as megalin, is a multiligand receptor expressed by macrophages in the liver (Kupffer cells) [36]. In a previous study, peritubular capillary damage and HFD-induced glomerular alterations were ameliorated in *LRP2*-knockout mice [17]. Another study reported that *LRP2* antagonized the attenuated effects of palmitate on clusterin-mediated insulin signaling and *APOA1* expression in human adipocytes and reduced hepatic gluconeogenesis in mice [37]. In this study, *LRP2* was found to be a target gene of *miR-380-5p* and downregulation of *LRP2* promoted lipid formation. Collectively, these results suggest the role of an *AC012668*/*miR-380-5p*/*LRP2* signaling pathway in the pathogenesis of NAFLD.

## Conclusion

In summary, the lncRNA *AC012668* was downregulated in NAFLD. Overexpression of *AC012668* suppressed lipid formation and protected against NAFLD by regulating the *miR-380-5p*/*LRP2* axis. These results may provide a promising strategy against NAFLD.

## Research highlights

1. miR-380-5p was verified to target AC012668
2. AC012668 expression was suppressed in NAFLD
3. LRP2 was confirmed to be the target gene of miR-380-5p
